# Ending the practice of corporal punishment in Nigeria: a call for progress and dignity

**DOI:** 10.1097/MS9.0000000000003582

**Published:** 2025-07-14

**Authors:** Chukwuka Elendu, Dependable C. Amaechi, Tochi C. Elendu, Deborah M. Olabode, Ijeoma D. Elendu, Emmanuel C. Amaechi, Ovonomor P. Aggreh, Chidera C. Bright

**Affiliations:** a Department of Medicine, Federal University Teaching Hospital, Owerri, Nigeria; b Department of Medicine, Igbinedion University, Okada, Nigeria; c Department of Nursing Science, Imo State University, Owerri, Nigeria; d Department of Medicine, Babcock University, Ilishan-Remo, Nigeria; e Department of Medicine, Madonna University, Elele, Nigeria; f Department of Medicine, Obafemi Awolowo College of Health Sciences, Ikenne, Nigeria; g Department of Medicine, University of Calabar, Calabar, Nigeria

**Keywords:** children’s rights, corporal punishment, digital platforms, international collaboration, positive parenting

## Abstract

Corporal punishment is a prevalent form of discipline in Nigerian schools, despite international and national efforts to protect children’s rights. Rooted in cultural norms and traditional practices, physical punishment is widely accepted by parents, educators, and community leaders as an effective disciplinary tool. It has remained deeply ingrained in Nigerian schools, with significant attention paid to its prevalence, detrimental effects, and the sociocultural factors that perpetuate its use. We conducted a literature review of 57 studies published over the past two decades, revealing alarmingly high rates of corporal punishment across different regions of Nigeria, with Northern Nigeria reporting the highest prevalence (85%) and Southern Nigeria the lowest (70%). Younger students, particularly those in primary school, are most frequently subjected to such disciplinary measures. Common forms include caning (60%), slapping (45%), and flogging (25%), all of which have significant physical, psychological, and emotional impacts – ranging from injuries to increased anxiety, depression, and low self-esteem. Traditional beliefs and societal attitudes normalize these practices, complicating the enforcement of protective laws such as the Violence Against Persons (Prohibition) Act of 2015. Our analysis underscores the pressing need for concerted efforts to eliminate corporal punishment in Nigerian schools. We recommend strengthening legal reforms, promoting alternative disciplinary approaches, engaging communities to shift cultural norms, and implementing effective monitoring mechanisms. By fostering positive discipline practices, Nigerian schools can create a safe and supportive environment that prioritizes the well-being and dignity of children.

## Public summary

The COVID-19 pandemic profoundly disrupted family dynamics and education systems globally, including in Nigeria. School closures and prolonged confinement at home likely increased the prevalence of corporal punishment, driven by heightened parental stress, financial strain, and the challenges of home-based learning. These circumstances intensified household tensions and may have led to greater reliance on punitive discipline. However, the pandemic also created opportunities for introspection as families spent more time together. Some parents – particularly those with access to online resources – began adopting more communicative and nonviolent disciplinary approaches.

**HIGHLIGHTS**

- Corporal punishment is highly prevalent, especially in northern Nigeria (85%).
- Common methods include caning, slapping, and flogging.
- Cultural norms and weak law enforcement sustain the practice.

Another major influence on corporal punishment practices in Nigeria is increased exposure to global perspectives through the internet and social media. As internet penetration deepens, platforms such as Twitter, Facebook, and Instagram have become powerful tools for advocacy. Campaigns like #EndCorporalPunishment have amplified the voices of survivors, parents, and educators, sparking dialogue and challenging traditional norms. These digital movements have raised awareness of the harms of corporal punishment and have prompted policy debates, urging the government and stakeholders to reconsider existing laws and disciplinary practices.

Internet access has also facilitated the dissemination of international research on child development and promoted alternative parenting strategies. Many Nigerian parents now engage with resources on positive reinforcement and conflict resolution, enabling them to adopt less punitive measures. Concurrently, children and adolescents – through social media – have become more aware of their rights and increasingly speak out against abuse.

Despite these promising developments, progress is uneven. Nigeria’s digital divide, particularly between rural and urban areas, limits the reach and impact of online advocacy. Deep-rooted cultural beliefs and the normalization of corporal punishment within communities continue to present major challenges. To build on current progress, coordinated efforts are needed – engaging policymakers, educators, religious leaders, and civil society – to expand awareness campaigns, promote parenting education, and enforce protective legislation.

The convergence of pandemic-related experiences, digital advocacy, and global awareness is reshaping societal attitudes toward corporal punishment in Nigeria. Sustained change, however, requires a comprehensive understanding of how historical patterns, evolving digital landscapes, and recent global crises have influenced disciplinary practices. Only through such insight can Nigeria move toward discipline methods that uphold children’s dignity, rights, and well-being.

## Introduction and background

Corporal punishment in educational settings remains a significant issue globally, particularly in developing countries like Nigeria. Defined as the deliberate infliction of physical pain as punishment, it reflects deep-rooted cultural norms, religious beliefs, and traditional disciplinary practices^[1]^. Despite international agreements and national laws protecting children’s rights, corporal punishment persists in Nigerian schools, raising serious concerns about its impact on students’ physical, psychological, and emotional well-being^[1–3]^. The practice is not only prevalent but also culturally ingrained. Many parents, educators, and community leaders perceive it as an effective tool for instilling discipline and respect^[4,5]^. This widespread acceptance complicates efforts to abolish the practice. Although the Violence Against Persons (Prohibition) Act of 2015 criminalizes various forms of violence, including corporal punishment, enforcement remains inconsistent^[6]^.

Studies highlight that teachers often justify its use as necessary for discipline, while cultural and traditional norms continue to legitimize the practice^[7,8]^. Research has linked corporal punishment to a range of harmful effects, including physical injuries, increased anxiety and depression, aggression, low self-esteem, and difficulty forming healthy relationships^[9–11]^. Sociocultural factors further reinforce its persistence, particularly in rural and conservative areas where traditional beliefs often support physical discipline. Weak enforcement, underreporting, and societal normalization of violence hinder the effectiveness of protective legislation^[12,13]^. These dynamics underscore the pressing need for culturally informed, systemic strategies to transform attitudes and practices. There is growing consensus among stakeholders – government, civil society, and NGOs – on the need to eliminate corporal punishment in schools. Interventions such as advocacy campaigns, teacher training, and community engagement initiatives are being pursued to promote positive disciplinary methods and foster safer learning environments^[14–16]^.

In line with current best practices for scientific integrity and the responsible integration of digital tools in research, we acknowledge the TITAN Guidelines 2025 for transparency in the reporting of artificial intelligence while noting that no generative AI tools were used in the preparation of our work^[17]^.

Our study synthesizes current evidence on the prevalence, types, impacts, and sociocultural drivers of corporal punishment in Nigerian schools. It aims to inform stakeholders and support evidence-based reforms that safeguard children’s rights and well-being^[18]^. In addition, we make a unique contribution to the existing literature by providing a context-specific synthesis that integrates cultural, legal, and public health perspectives on corporal punishment in Nigerian schools. Unlike previous works that focus narrowly on either legal frameworks or psychological outcomes, our study offers a holistic analysis that highlights the intersectionality of sociocultural beliefs, policy gaps, and child rights advocacy.

Key learning points emerging from this work include: (1) the normalization of corporal punishment is deeply rooted in cultural and religious beliefs; (2) enforcement of existing protective laws remains weak; (3) corporal punishment has wide-ranging psychological and developmental impacts; and (4) a multisectoral, culturally tailored approach is essential to achieving sustainable change.

## Data collection

The inclusion criteria for the studies were as follows: (1) focus on corporal punishment in Nigerian educational settings, (2) publication in peer-reviewed journals, (3) studies conducted within the last 20 years to ensure contemporary relevance, (4) articles written in English, and (5) qualitative or quantitative research examining the prevalence, impacts, sociocultural factors, or interventions related to corporal punishment.

The exclusion criteria were: (1) studies not specific to Nigeria, (2) articles not peer-reviewed, (3) studies published before the year 2000, (4) non-English language publications, and (5) studies that did not address corporal punishment directly or tangentially.

A structured search strategy was implemented using electronic databases, including PubMed, PsycINFO, ERIC (Education Resources Information Center), Scopus, and Google Scholar. Keywords and search terms included combinations of “corporal punishment,” “physical discipline,” “Nigeria,” “schools,” “students,” “prevalence,” “impacts,” “cultural factors,” “legislation,” and “policy.” Boolean operators (AND, OR) were applied to refine results and ensure broad yet targeted retrieval.

**Figure 1.**
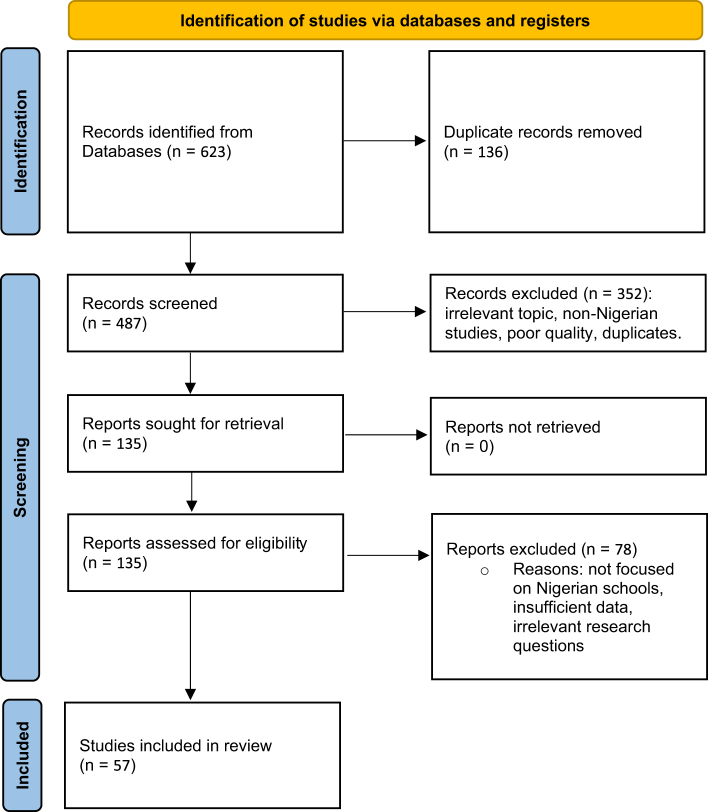
PRISMA flow diagram illustrating the process of study selection.

The initial search yielded 623 articles, reduced to 487 unique entries after removing duplicates. Two independent reviewers screened the titles and abstracts to assess relevance to the study’s aims. This process excluded 352 articles that did not meet the inclusion criteria. The remaining 135 articles underwent full-text review, with 78 excluded due to a lack of specific focus on Nigerian schools, irrelevant research questions, or insufficient data on corporal punishment. Ultimately, 57 studies were included for detailed analysis, as illustrated in the PRISMA flow diagram (Fig. 1) and summarized in Table 1.

The PRISMA flow diagram depicts the study selection process, outlining the identification, screening, eligibility assessment, and inclusion of studies considered in this review. Records were excluded for reasons including non-Nigerian focus (*n* = 27), insufficient outcome data (*n* = 18), poor methodological quality (*n* = 12), previously undetected duplicates (*n* = 9), and language other than English (*n* = 12). Source: Authors’ creation.

**Table 1:** Summary of key studies on corporal punishment in Nigerian schools

Author(s) and year	Region/Scope	Study design	Key findings	Relevance to corporal punishment
[Fakunmoju SB, 2020]	National	Survey-based study	Majority of teachers still perceive corporal punishment as necessary	Highlights persistent educator support for physical discipline
[Bammeke and Fakunmoju, 2016]	National	Qualitative study	Cultural and traditional beliefs strongly influence disciplinary practices	Shows deep-rooted sociocultural justification for corporal punishment
[Gershoff ET, 2017]	Nationwide (Regional breakdown)	Meta-analysis	Prevalence rates: 85% (North), 83% (East), 78% (West), 75% (South)	Quantifies the widespread use of corporal punishment
[Heekes SL et al., 2022]	National	Systematic review	Most common methods: caning (60%), slapping (45%), whipping (30%), flogging (25%), kneeling (10%)	Illustrates diverse and prevalent forms of physical punishment
[Fakunmoju and Bammeke, 2015; Oates, 2011; Durrant, 2008]	National	Review and cross-sectional studies	Reported effects: physical injuries, anxiety, depression, aggression, low self-esteem	Emphasizes the harmful psychological and emotional impacts
[Esezobor and Balogun, 2016; Adeleye et al., 2023]	Rural and conservative regions	Sociocultural analysis	Strong societal support for physical discipline as norm	Explains enforcement challenges despite legal bans
[Global Partnership, 2019; WHO, 2020; Sanders, 1999]	National (policy scope)	Program evaluations	Ongoing efforts: advocacy, teacher training, positive discipline campaigns	Demonstrates growing momentum for reform and intervention

The selected studies underwent thematic analysis to identify patterns related to the prevalence, impacts, and sociocultural drivers of corporal punishment. Thematic analysis was chosen for its flexibility and ability to organize and describe data in rich detail systematically. Two reviewers independently conducted open coding by reading and annotating full texts. Initial codes were generated inductively and grouped into broader categories. These categories were refined into overarching themes, including prevalence, psychological impacts, and cultural drivers. The coding process was iterative, with themes adjusted as new insights emerged. Discrepancies were resolved through discussion, and a third reviewer was consulted when necessary to ensure consistency and accuracy.

To ensure replicability, detailed records of the search strategy, including keywords, databases, and search dates, were maintained. The search was conducted between January and March 2024. Specific search terms included “corporal punishment Nigeria,” “physical discipline in Nigerian schools,” “impact of corporal punishment on Nigerian students,” and “cultural acceptance of corporal punishment in Nigeria.” Boolean operators and truncation symbols were used to facilitate replication. Throughout the data extraction and analysis process, two reviewers independently assessed each study to minimize bias and enhance the reliability of the results. Discrepancies were resolved through discussion and, where needed, consultation with a third reviewer. This rigorous approach ensured the credibility and robustness of the review’s findings.

## Results

Our study included 57 studies that met the eligibility criteria and provided relevant insights into the prevalence, types, and impacts of corporal punishment in Nigerian schools. The combined analysis revealed that corporal punishment remains a widespread disciplinary method across Nigerian educational institutions, with prevalence rates varying significantly by region and educational level. Northern Nigeria reported the highest prevalence (85%), followed by Eastern (80%), Western (75%), and Southern Nigeria (70%), as detailed in Table 2, which outlines prevalence by region, age group, and type of punishment.

**Table 2:** Summary of prevalence and types of corporal punishment in Nigerian schools

Geographic region	Percentage of schools using corporal punishment	Age group	Types of punishment	Demographics
Northern Nigeria	85%	Primary	Caning, Slapping	Low-income families, marginalized communities
		Secondary	Whipping, Flogging	Children with disabilities
		Tertiary	Other methods	Gender disparities
Southern Nigeria	70%	Primary	Caning, Slapping	Low-income families
		Secondary	Slapping, Flogging	Marginalized communities
		Tertiary	Caning, Whipping	Gender disparities
Western Nigeria	75%	Primary	Caning, Slapping	Low-income families
		Secondary	Slapping, Whipping	Children with disabilities
		Tertiary	Caning, Flogging	Marginalized communities
Eastern Nigeria	80%	Primary	Caning, Slapping	Low-income families, marginalized communities
		Secondary	Slapping, Flogging	Gender disparities
		Tertiary	Caning, Whipping	Children with disabilities

Fifty-seven studies outline the prevalence and types of corporal punishment in Nigerian schools. Notably, Northern Nigeria shows the highest prevalence at 85%. Primary school students are particularly affected, with caning identified as the most common form of punishment (60%). Other prevalent forms include slapping (45%), whipping (30%), and flogging (25%), highlighting the pervasive use of physical discipline across different educational levels. Source: Authors’ Creations.

Across age groups, primary school students experienced the highest rates of corporal punishment – 80% in Northern Nigeria, 75% in Southern, 78% in Western, and 83% in Eastern regions. Secondary school students reported lower prevalence rates (65% in the North, 60% in the South, 68% in the West, and 70% in the East). Tertiary-level students had the lowest rates, ranging from 40% to 50% across regions.

A variety of corporal punishment methods were reported. Caning was the most common (cited in 60% of the studies), followed by slapping (45%), whipping (30%), and flogging (25%). Less common methods – such as kneeling, prolonged standing, and other physically straining punishments – were identified in 10% of the studies.

**Figure 2.**
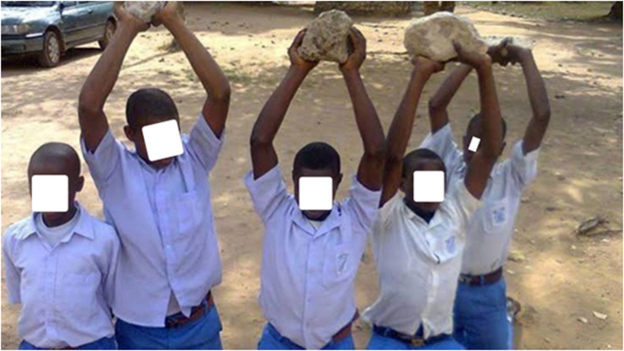
Instances of corporal punishment in Nigerian schools.

**Figure 3.**
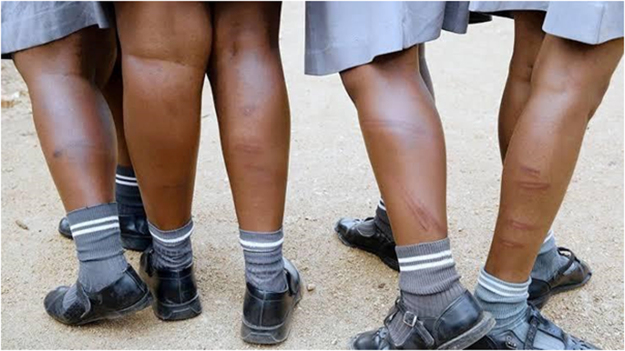
Aftermath of corporal punishment on female students.

**Figure 4.**
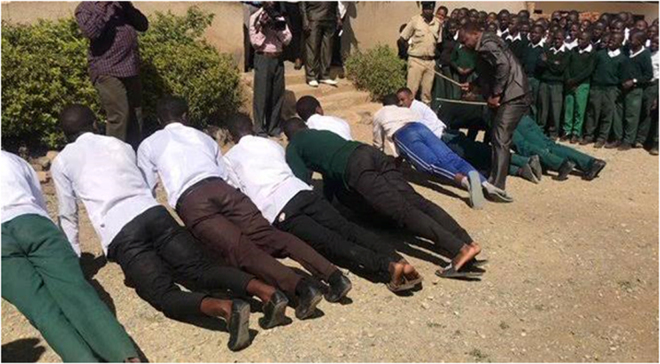
Principal administering corporal punishment for tardiness.

**Figure 5.**
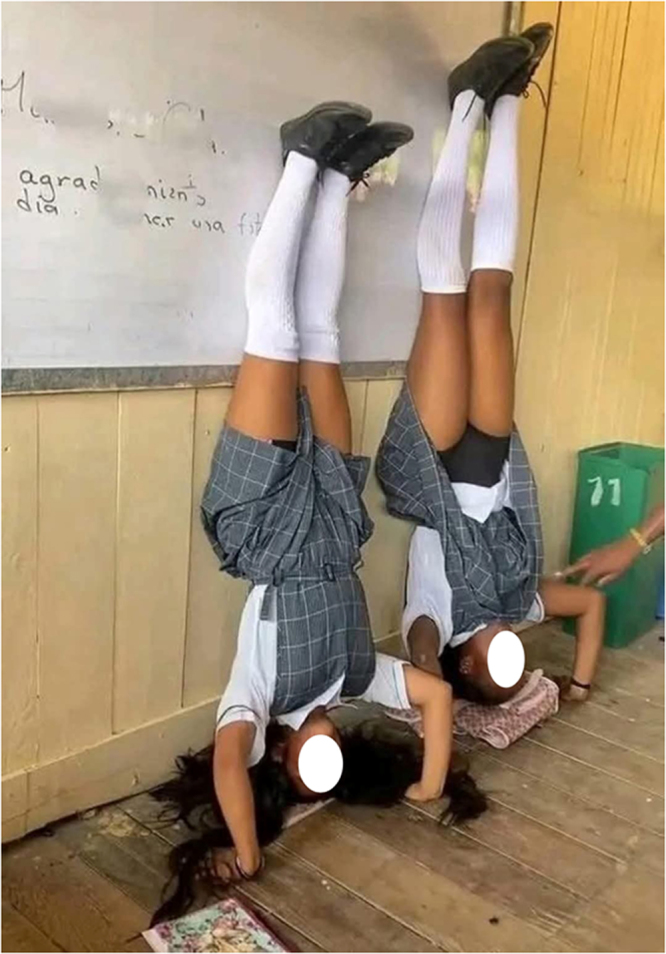
Students punished for lateness in an inverted and physically straining position.

Multimedia evidence from several studies further underscores the severity and normalization of these practices. Figure 2 depicts students forced to kneel under the sun while carrying blocks – a punitive measure still practiced in some schools. Supplemental Digital Content Video 1, available at: http://links.lww.com/MS9/A884 presents a real-time classroom scene that illustrates the application of physical discipline, highlighting its normalization and intensity. Figure 3 shows lash marks on female students’ legs following flogging for speaking native languages. Figure 4 captures a school principal administering corporal punishment to latecomers. In contrast, Figure 5 shows students subjected to an unnatural, physically straining punishment posture, with their heads and hands on the floor and legs raised. These visuals emphasize the physical and psychological toll of such disciplinary practices.

Regarding the impact of corporal punishment, studies reported a wide range of adverse outcomes. Physical effects included bruises, welts, and other injuries. Psychological consequences such as anxiety, depression, aggression, and post-traumatic symptoms were frequently documented. Emotional outcomes included low self-esteem and difficulty forming trusting relationships.

Sociocultural factors that perpetuate corporal punishment were also identified. These include cultural beliefs that emphasize obedience, perceptions that physical discipline is effective, and normalization of violence – particularly in rural and conservative regions.

## Discussion

Scholars trace the roots of corporal punishment in Nigeria to traditional African societies where communal values and hierarchical structures legitimized physical discipline to maintain order and social norms^[1–3]^. These practices were reinforced during the colonial era, as British administrators institutionalized corporal punishment in schools and legal systems to assert control^[4–6]^. Following independence in 1960, Nigeria retained many colonial-era disciplinary systems, with subsequent authoritarian governments further entrenching the use of force in public institutions^[19,20]^. In recent years, both local and international advocacy efforts have intensified. Organizations like the Nigerian Human Rights Commission and the Nigerian Association for the Protection of Children have led legislative reform and awareness campaigns^[21,22]^. Supported by frameworks such as the UN Convention on the Rights of the Child, these initiatives emphasize the need to protect children from violence, including corporal punishment^[11,23]^.

Despite these efforts, corporal punishment remains prevalent in Nigerian schools. Northern Nigeria reports the highest incidence, while the South reports the lowest. Younger students are disproportionately affected. While caning is most commonly used, slapping, whipping, and flogging are also frequent, with some cases escalating into abuse and serious harm^[24–26]^.

The impact of corporal punishment is multifaceted. Physically, it can lead to injuries such as bruises and long-term musculoskeletal problems, especially with repeated use of objects like canes^[3–5]^. Psychologically, it induces fear, anxiety, and mistrust, eroding students’ sense of safety^[6,7]^. Emotionally, it contributes to shame, depression, PTSD, and emotional dysregulation^[8–10]^. These outcomes often lead to behavioral issues like aggression and antisocial tendencies, which disrupt learning^[11,23,27]^. Corporal punishment may also normalize violence, hinder empathy, and diminish academic performance^[12,28–30]^.

Sociocultural beliefs, gender norms, religious teachings, and power dynamics continue to support corporal punishment in schools^[1–4]^. Although Nigeria has ratified international treaties such as the UN Convention on the Rights of the Child (1991) and the African Charter on the Rights and Welfare of the Child, domestic enforcement is weak. National laws, such as the Child Rights Act (2003) and the Violence Against Persons (Prohibition) Act (2015), prohibit corporal punishment; however, public awareness and institutional accountability remain limited^[3–6]^. Bridging this gap requires enhanced public education, educator training, and enforcement mechanisms.

Alternative disciplinary models are gaining ground. These include restorative justice, Positive Behavior Support (PBS), and trauma-informed practices^[8–10]^. Restorative justice involves peer mediation and dialogue to build empathy. PBS promotes clear expectations, reinforces positive behavior, and fosters emotional self-regulation. Trauma-informed approaches and social-emotional learning focus on students’ personal histories and aim to improve peer relationships and reduce violence^[3,4,11,23]^.

Corporal punishment infringes on children’s rights to dignity, safety, and education. Article 19 of the Convention on the Rights of the Child bans all forms of violence, while Articles 28 and 29 support inclusive education that fosters well-being. Inflicting pain and humiliation undermines these principles, creating hostile school environments^[1,2]^.

Corporal punishment also promotes a culture of aggression by presenting violence as an acceptable form of control. It compromises classroom safety and learning. Gender-based disparities are notable: boys often receive harsher punishments due to societal expectations of masculinity^[5,6]^, while girls may face verbal and psychological discipline reinforcing submission^[4,5]^. Male teachers tend to discipline boys more strictly, often framing it as preparation for adult responsibilities^[25]^. These double standards highlight the need for gender-sensitive training and equitable discipline policies.

Marginalized students face additional challenges. Children from low-income families often attend under-resourced schools where corporal punishment is more prevalent^[1-3]^. Students with disabilities are disproportionately affected due to ableist stereotypes^[4,5]^. Ethnic minority students may experience discriminatory treatment based on cultural bias^[6,7]^. These intersecting forms of inequality must be addressed.

Global examples offer useful models. Countries like Sweden, Finland, and New Zealand have banned corporal punishment and implemented caregiver education and training programs^[1,2]^. These systems promote communication, respect, and nonviolence. Adapting such approaches in Nigeria requires local customization. Collaboration among government, NGOs, educators, parents, and communities is essential. Grassroots initiatives, including anti-bullying programs and youth advocacy, have shown promise in changing social norms^[5,6]^. International collaborations, such as with the Global Partnership to End Violence Against Children, further bolster these efforts through funding, training, and advocacy^[7]^.

Religious teachings have historically justified corporal punishment. However, modern theological interpretations increasingly support nonviolent parenting. Christian groups, such as the World Council of Churches, and Muslim scholars, including Sheikh Abdullah Bin Bayyah, promote compassionate discipline^[1–4]^. Other religions, including Buddhism, Hinduism, and Judaism, emphasize child protection. Interfaith coalitions, such as the Global Network of Religions for Children, advance advocacy for nonviolent discipline^[6–8]^.

Media and popular culture in Nigeria often depict corporal punishment as a legitimate disciplinary measure. Nollywood films, songs, and proverbs reinforce these views. This exposure fosters desensitization and acceptance of violence. Media literacy programs are essential for challenging these narratives and promoting respectful conflict resolution. Responsible storytelling can change public perceptions. Government officials must take an active role in prevention, enforcement, and support for victims.

Several grassroots programs demonstrate successful intervention. The Society for the Improvement of Rural People (SIRP) has led positive discipline campaigns in rural areas, such as Ikot Udo Nkan, which have reduced violence and improved family dynamics^[8]^. Parent mentoring and support groups have helped caregivers adopt nonviolent approaches, enhancing child safety and academic performance^[10]^. The Family Life and Gender Education Foundation (FLAGEF) in Jos has worked with schools, religious institutions, and local leaders through its “Parenting Without Violence” campaign. FLAGEF has helped schools ban corporal punishment, train educators in emotional intelligence, and foster respectful learning environments^[26,31]^. Addressing corporal punishment in Nigeria requires a coordinated, multi-stakeholder approach through the following streamlined, actionable recommendations that promote sustainable change:

- Legislative Reforms: Strengthen enforcement of laws prohibiting corporal punishment and align national legislation with global standards. Raise public awareness and ensure accountability for violations^[1]^.
- Teacher Training: Offer ongoing professional development that focuses on positive reinforcement, effective communication, and conflict resolution^[2]^.
- Community Engagement: Foster dialogue with parents, religious leaders, and stakeholders to challenge cultural norms and promote empathy-based parenting^[3]^.
- Capacity Building: Empower NGOs and civil society with funding, training, and partnerships to scale up advocacy and monitoring initiatives^[4]^.
- Public Awareness: Launch inclusive, culturally sensitive campaigns via television, radio, and social media to educate the public on the harms of corporal punishment and promote positive alternatives^[32]^.

## Concluding remarks

Corporal punishment in Nigeria constitutes a serious violation of children’s rights and poses significant risks to their physical, emotional, and psychological development. This paper has examined the wide-ranging impacts of corporal punishment on children, families, and society, underscoring the urgent need for reform and the adoption of alternative, nonviolent disciplinary practices rooted in positive parenting and respect for human dignity.

By examining the historical context, cultural drivers, and global best practices, it is evident that eliminating corporal punishment necessitates a coordinated, multisectoral response. Legislative enforcement, public education, professional training, and community-based interventions are essential components of a sustainable solution. With sustained advocacy and investment in research and capacity-building, Nigeria can create a safer and more nurturing environment for all children.

Moreover, by engaging international partners and leveraging digital platforms, Nigeria can access critical resources, technical support, and global solidarity to accelerate progress. Through strategic collaboration and shared responsibility, we can work toward a society where every child is protected, empowered, and free from violence.

## Call to action

Let us reaffirm our collective responsibility to build a Nigeria where corporal punishment is no longer tolerated and where every child is allowed to grow, learn, and thrive in safety and dignity. We urge policymakers, educators, parents, faith leaders, and civil society to join us in this commitment. Together, we can contribute to the global movement to end all forms of violence against children – and ensure a brighter, more compassionate future for generations to come.

## Data Availability

All data generated or analyzed during our study are included in this published version. All data generated or analyzed during this study are included in this published article and its supplementary information files.

## Research registration unique identifying number (UIN)

N/A.
